# Supplementary material for: Asymmetric regeneration dynamics in zebrafish pectoral fins
Source: bioRxiv. 2026 Oct 28:2026.10.26.761995. Preprint. [Version 1] doi: 10.64898/2026.10.26.761995 (PMC13733998; doi:10.64898/2026.10.26.761995)

bioRxiv preprint doi: https://doi.org/10.64898/2026.10.26.761995; this version posted October 28, 2026. The copyright holder for this preprint (which was not certified by peer review) is the author/funder, who has granted bioRxiv a license to display the preprint in perpetuity. It is made available under a CC-BY 4.0 International license.

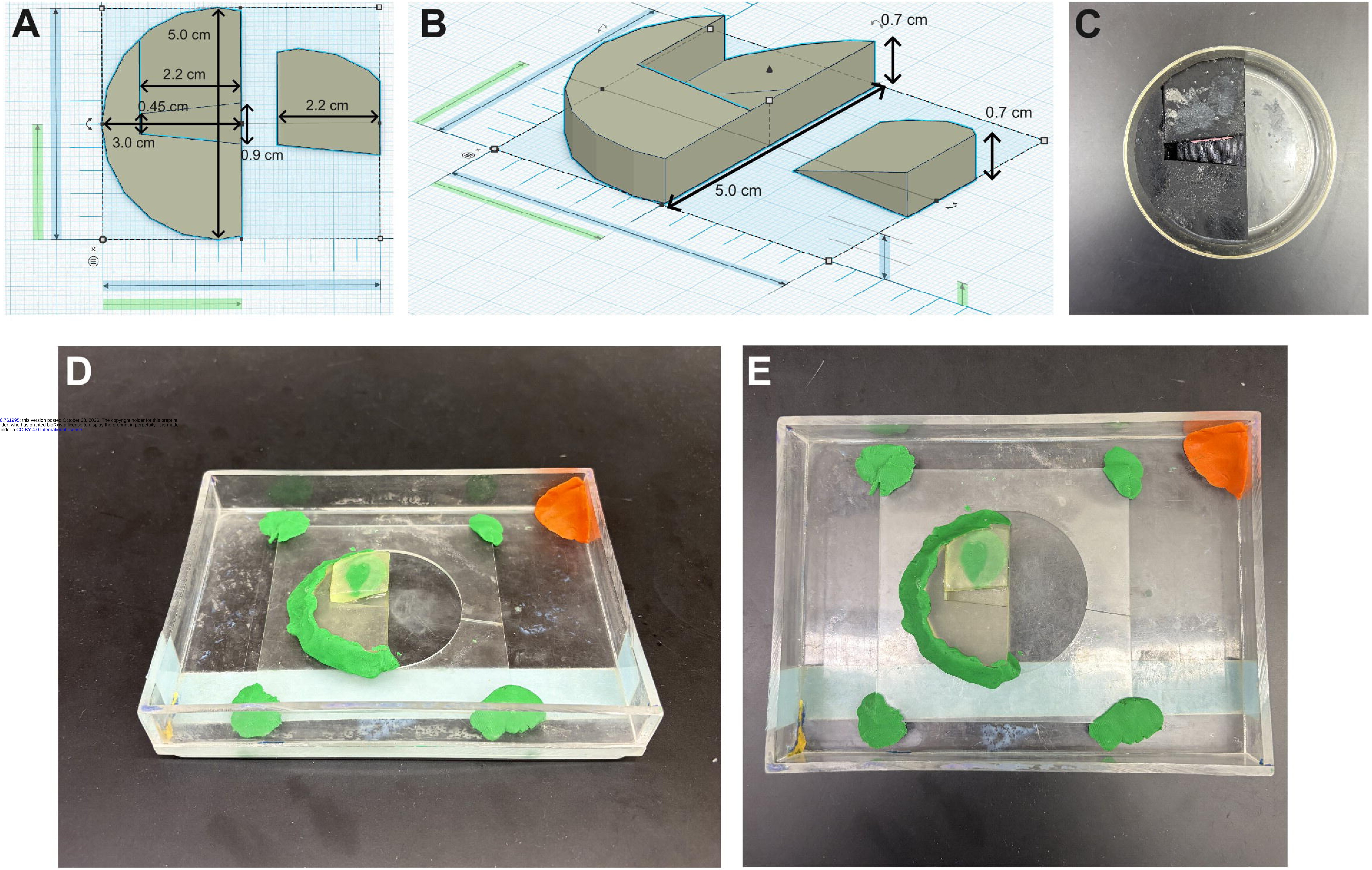

bioRxiv preprint doi: https://doi.org/10.64898/2026.10.26.761995; this version posted October 28, 2026. The copyright holder for this preprint (which was not certified by peer review) is the author/funder, who has granted bioRxiv a license to display the preprint in perpetuity. It is made available under a CC-BY 4.0 International license.

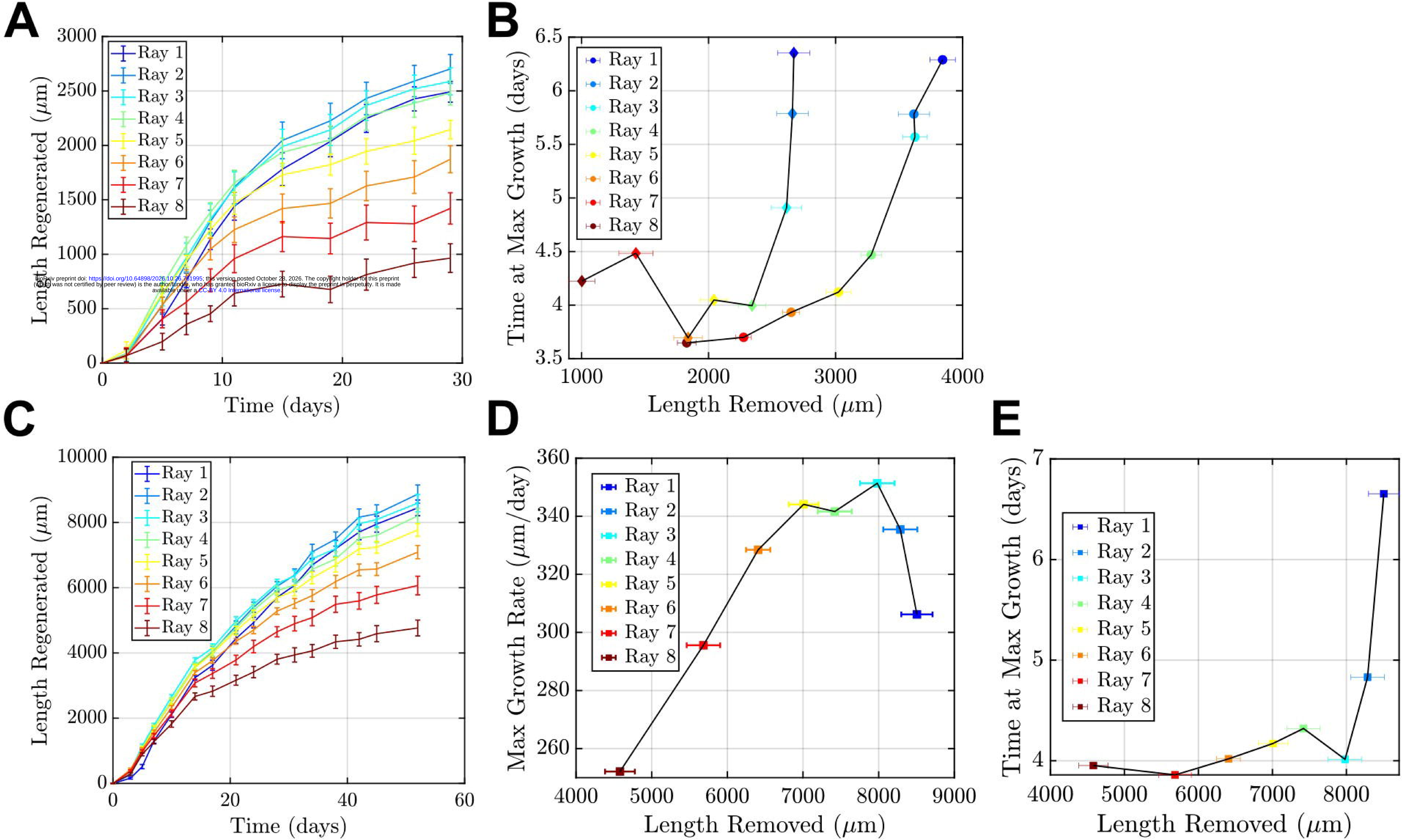

Supplement: Supplementary file 1 [file NIHPP2026.10.26.761995V1-supplement-1.pdf]
